# Image-Guided Intensity-Modulated Radiation Therapy for IgG4-Related Ophthalmic Disease

**DOI:** 10.1155/2020/8873078

**Published:** 2020-09-05

**Authors:** Rachel A. Sabol, Virginia Bubenzer, Krzysztof Moroz, Shams Halat, Audrey Dang, Keith Ferdinand, Angela Traylor, Carol Boyd, Kendra Harris

**Affiliations:** ^1^Tulane University School of Medicine, New Orleans, LA, USA; ^2^Department of Radiation Oncology, Tulane University School of Medicine, New Orleans, LA, USA; ^3^Department of Pathology, Tulane University School of Medicine, New Orleans, LA, USA; ^4^Department of Cardiology, Tulane University School of Medicine, New Orleans, LA, USA; ^5^Department of Psychiatry and Neurology, Tulane University School of Medicine, New Orleans, LA, USA

## Abstract

**Background:**

IgG4-related ophthalmic disease is a rare, newly recognized entity with high failure rates on first-line therapy of systemic corticosteroids and no other proven management options. *Case Presentation.* Here, we present the clinical course of a patient with IgG4 ophthalmic disease who achieved a favorable response from radiotherapy. Our patient initially presented with a history of recurrent painful flares of orbital inflammation, a pathologic diagnosis follicular lymphoid hyperplasia from a right lacrimal gland biopsy, and MRI imaging noting expansion of the lateral rectus muscle of the right eye. Initial treatment with dacryoadenectomy and multiple courses of corticosteroids failed to keep his symptoms at bay. Further evaluation revealed florid IgG4 staining. In this context, he was evaluated for image-guided intensity-modulated radiotherapy (IG-IMRT) to the orbit to 20 Gy in 10 fractions. His ophthalmic symptoms resolved.

**Conclusions:**

This treatment experience suggests radiotherapy may be a favorable option for symptom relief in patients with IgG4-related ophthalmic disease not controlled by corticosteroids.

## 1. Introduction

IgG4-related ophthalmic disease (IgG4-ROD) is a relatively recently recognized ocular inflammatory entity characterized by the infiltration of IgG4-positive plasma cells into tissues, typically ocular muscles. The etiology and pathogenesis of this process is currently unknown [1, 2]. IgG4-ROD can present as an inflammation limited to the orbit or can present as a systemic sclerosing inflammatory condition that can affect many different organs including the orbit, pancreas, liver, kidney, lung, and thyroid [3–5]. IgG4-ROD may be responsible for a significant proportion of what had previously been labeled idiopathic orbital inflammation/reactive lymphoid hyperplasia and should be in the differential for patients found to have ocular adnexal inflammation [2, 6–8].

Current treatments for IgG4-ROD limited to the orbit are guided by systemic agents used to address extraorbital multifocal disease. First-line therapy is often systemic corticosteroids, but retrospective data suggests progression/relapse rates as high as 72% [9]. Other systemic treatments for which there are published case series data include rituximab and other immunosuppressants such as azathioprine, methotrexate, mycophenolate, and cyclosporine [10]. Radiation has been described in the context of primary therapy and recurrent disease; however, variable responses have been described [7, 11–13].

Here, we describe the clinical course of a patient found to have IgG4-ROD, treated with image-guided intensity-modulated radiation therapy (IG-IMRT) who had an excellent and rapid clinical response at 3-month follow-up.

## 2. Case Presentation

**Figure 1:**
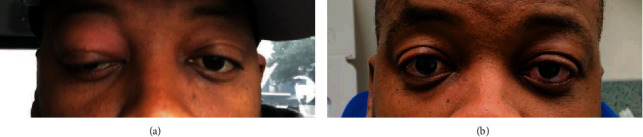
(a) Patient with orbit inflammation after sugery and multiple rounds of steroids prior to radiation. (b) Follow-up: 3 months after radiation with resolution of inflammation and symptomatic improvement.

The 39-year old patient initially presented in 2017 for recurrent episodes of dry, painful, swelling of the right eye (Figure 1(a)). Computerized tomography (CT) images at that time demonstrated enlargement of the right lacrimal gland and increased size of the right lateral rectus muscle. The patient underwent right orbitotomy with tissue exploration with a biopsy of the posterior right lateral rectus muscle, right dacryoadenectomy, and right temporal tarsorrhaphy. Gross examination noted an abnormally hard lacrimal gland and pathology revealed intraglandular follicular lymphoid hyperplasia with no phenotypically abnormal lymphocytes on flow cytometric analysis. Pathology of the lateral recuts also demonstrated intramuscular follicular lymphoid hyperplasia. Of note, this patient's past medical history is significant for eosinophilic esophagitis without other known history of autoimmune diseases, and he is followed by cardiology for malignant hypertension treated with multiple antihypertensive agents. He denies ever having experienced unexplained weight loss, night sweats, or unexplained fevers.

**Figure 2:**
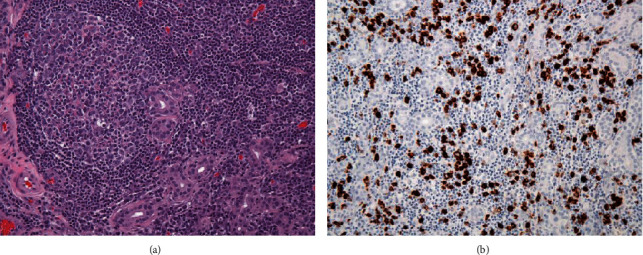
(a) Lacrimal gland obliterated by dense lymphoplasmacytic infiltrate containing reactive lymphoid aggregate (H&E, 200x). (b) IgG4 immunohistochemical stain showing abundant IgG4+ plasma cells (IgG4, 200x).

The patient received dozens of courses of oral steroids over the course of the two years prior to presenting to radiation oncology. The steroid courses were of varying doses and duration and were not well tolerated due to medical comorbidities, mainly malignant hypertension. In 2019, he began to experience increased severity of symptoms during flares, rating pain 10/10 with no response to systemic steroids. Symptoms included new onset diplopia, severe ocular pain, and worsening upper and lower palpebral edema described by the patient as intermittently “so bad I can't open my eye at all.” His physical exam was significant for mild right proptosis, right-ptosis, evoked diplopia with inability to fully medialize the right globe, and incomplete conjugate gaze at the far medial and lateral fields without appreciable impairment of visual acuity. The patient was referred to radiation oncology, where his constellation of findings prompted consideration of the possibility of IgG4-ROD and a request for IgG4 staining of tissue from his 2017 orbitotomy was sent and shown to be 100 per high-powered field (Figure 2). A number of blood tests were also ordered: EBV-, HIV-, and CMV-; quantitative systemic immunoglobulins (G, M, A) were all within the normal range.

Treatment options were reviewed. In the context of his comorbidities and polypharmacy, radiotherapy was selected as the preferred treatment approach by the patient over other second-line immunomodulators that have been described for use in IgG4-ROD to avoid the addition of another systemic therapy. We discussed that IgG4-ROD is one of a number of benign inflammatory conditions of the orbit including graves ophthalmopathy, orbital pseudotumor, and precursor lymphoproliferative process (up to and including lymphomas, in particular, mucosa-associated lymphoid tissue (MALT) lymphomas). We discussed that low-dose radiotherapy can be given to address the symptoms in the context of these other orbital processes and discussed the risks and benefits of a course of IG-IMRT with the goal of symptom control. After informed consent was obtained, the patient underwent IG-IMRT of 20 gray (Gy) in 10 fractions to the right orbit with attention to sparing the brain and lens.

Although he experienced an inflammatory flare during the first week of radiotherapy, he subsequently had an impressive clinical response (Figure 1). In the three months since treatment, no further orbital inflammatory flares have occurred and repeat imaging with orbital MRI confirms radiographic response. While 3-month follow-up is short to assess recurrence, it is notable in the context of symptom resolution and quality of life improvement reported by the patient.

## 3. Methods

### 3.1. Radiation Course

The patient was immobilized with a short aquaplast mask and underwent a CT simulation without IV contrast and an orbital MRI with gadolinium. The treatment field was defined as the bony orbit. An intensity-modulated radiotherapy plan with volumetric modulated arc radiotherapy (V-MAT) using 6 MV photos was created with two arcs delivered with the couch at 0 degrees and one arc delivered with the couch at 280 degrees. The quality of the plan was assessed for conformality and homogeneity. Daily image-guidance with cone beam CT was undertaken with a match to the bone. The dose was 20 Gy in 10 fractions.

## 4. Discussion

IgG4-ROD typically presents with fibro-inflammation of the orbit and symptoms of unilateral or bilateral proptosis, eyelid swelling, ocular pain, and impaired ocular movement [9, 10]. Other parts of the orbit, such as the extraocular muscles, orbital adipose and soft tissue, infraorbital nerve and canal, and nasolacrimal duct system, can be involved with key distinguishing features including a disproportionate involvement of the lateral rectus muscle and a typical tendon-spring morphology as well as lid retraction/lid lag [7, 10, 14]. The majority of published cases have described patients of Asian descent, and there is some controversy in the literature over the occurrence of IgG4-ROD in Western patients and if conclusions drawn from patients in Asia can be extrapolated more generally [8]. The clinic course of our patient contributes to the growing body of literature of IgG4-ROD in Western patients and offers evidence of a therapeutic response to low-dose radiotherapy.

Therapeutic options are important for this cohort of patients as recurrent symptoms are common and long-term steroids do not always produce the desired clinical relief. Additionally, inadequate steroid dose can lead to insufficient immunosuppression, and as was seen in this case, steroids could not be tolerated due to a history of malignant hyperthermia treated with multiple antihypertensives. Even when steroids do provide relief, long-term corticosteroid use is associated with many negative side effects including osteoporosis, hyperlipidemia, hyperglycemia, and adrenal insufficiency [15]. When steroids cannot be tolerated, and/or there is diminishing relief provided by steroids, alternative treatment options must be considered. In IgG4-ROD, immunomodulators including Rituximab are often considered as the second-line therapy. Additionally, radiation has been described in case reports as primary treatment, adjuvant therapy, or in combination with steroids and/or Rituximab, with variable responses and reported outcomes [7, 11–13, 16].

Given the variety of clinical scenarios in which radiotherapy has been used, as well as variable reported efficacy, this case report provides an important and unique perspective [11, 12]. This case adds an important additional perspective to the literature regarding IgG4-ROD describing symptom improvement and short-term response following 20 Gy radiotherapy. There are published experiences of treating IgG4-ROD with radiation; however, these reports demonstrate a variety of responses to radiation. Thus, there is a critical need to share our case report and experience to expound on other cases so clinicians can evaluate the body of experiences as a whole for clinical decision-making [11]. Here, we present an additional experience that expands upon and strengthens the growing body of literature by describing a patient with symptom improvement and short-term response to radiotherapy.

While there have been case reports of the use of radiotherapy in IgG4-ROD, the toxicities are often not described in the context of clinical decision-making in these cases. One of the most common side effects of radiotherapy to the orbit is dry eye syndrome. In this case, the patient had previously had a dacryoadenectomy in the affected eye that was to be treated with radiotherapy, which helped guide decision-making as the patient already had iatrogenically induced dry eye syndrome due to previous surgery. Radiation is often reserved as a second line therapy due to the increased risk of secondary malignancy following radiation.

Additionally, this case demonstrates a diagnosis of IgG4-ROD two years after initial presentation. In the time between initial surgery in 2017 and definitive diagnosis, the patient continued to receive many courses of oral steroids and experienced adverse effects until steroids were no longer tolerable. As a relatively new pathology, it is expected that more cases of IgG4-ROD will be diagnosed after several therapies have been initiated. Understanding the role that radiotherapy can play in these cases provides a valuable addition to the literature and provides treatment experience that may apply to patients diagnosed under similar circumstances.

Without available prospective data, case reports are an important way to prompt our colleagues to consider IgG4-ROD when evaluating patients with inflammatory orbital disease. As with all newly described entities, given a tool to assess for IgG4 staining, the number of patients who can be assessed as having IgG4-ROD will rise and, with it, a desire for data related to treatment options. This case report provides an important perspective in that the diagnosis of IgG4-ROD was made several years after initial evaluation and attempted disease management with steroids that were not well tolerated. Consideration of low-dose radiotherapy may be reasonable in the right clinical circumstance.

## Data Availability

Data sharing is not applicable to this article as no datasets were generated or analyzed during the current study.

## Abbreviations

(IgG4-ROD): IgG4-related ophthalmic disease
(IG-IMRT): Image-guided intensity-modulated radiation therapy
(CT): Computerized tomography
(MRI): Magnetic resonance imaging
(MALT): Mucosa-associated lymphoid tissue
(Gy): Gray.
